# Pathogenic, but Not Nonpathogenic, *Rickettsia* spp. Evade Inflammasome-Dependent IL-1 Responses To Establish an Intracytosolic Replication Niche

**DOI:** 10.1128/mbio.02918-21

**Published:** 2022-02-08

**Authors:** Oliver H. Voss, Jennifer Cobb, Hodalis Gaytan, Natalia Rivera Díaz, Rigoberto Sanchez, Louis DeTolla, M. Sayeedur Rahman, Abdu F. Azad

**Affiliations:** a Department of Microbiology and Immunology, University of Maryland School of Medicine, Baltimore, Maryland, USA; b Department of Pathology, University of Maryland School of Medicine, Baltimore, Maryland, USA; c Department of Medicine, Division of Infectious Diseases, University of Maryland School of Medicine, Baltimore, Maryland, USA; d Department of Epidemiology and Public Health, University of Maryland School of Medicine, Baltimore, Maryland, USA; Ohio State University

**Keywords:** *R. typhi*, *R. rickettsii* Sheila Smith, *R. montanensis*, IL-1α, IL-1β, caspase-1, caspase-11, macrophages, inflammasomes

## Abstract

*Rickettsia* species (spp.) are strict obligate intracellular bacteria, some of which are pathogenic in their mammalian host, including humans. One critical feature of these stealthy group of pathogens is their ability to manipulate hostile cytosolic environments to their benefits. Although our understanding of *Rickettsia* cell biology and pathogenesis is evolving, the mechanisms by which pathogenic *Rickettsia* spp. evade host innate immune detection remain elusive. Here, we show that disease severity in wild-type (WT) C57BL/6J mice infected with Rickettsia typhi (the etiologic agent of murine typhus) and Rickettsia rickettsii (the etiologic agent of Rocky Mountain spotted fever), but not with the nonpathogenic species Rickettsia montanensis, correlated with levels of bacterial burden as detected in the spleens of mice, as well as the serum concentrations of proinflammatory cytokine interleukin-1α (IL-1α) and, to a lesser extent, IL-1β. Antibody-mediated neutralization of IL-1α confirmed a key role in controlling mortality rates and bacterial burdens of rickettsia-infected WT mice. As macrophages are a primary source of both IL-1α and IL-1β cytokines, we determined the mechanism of the antirickettsial activities using bone marrow-derived macrophages. We found that pathogenic R. typhi and R. rickettsii, but not nonpathogenic R. montanensis, eluded pro-IL-1α induction and benefited predominantly from the reduced IL-1α secretion, via a caspase-11–gasdermin D (Gsdmd)-dependent pathway, to facilitate intracytosolic replication. Adoptive transfer experiments identified that IL-1α secretion by macrophages was critical for controlling rickettsiosis in WT mice. In sum, we identified a previously unappreciated pathway by which pathogenic, unlike nonpathogenic, rickettsiae preferentially target the caspase-11–Gsdmd–IL-1α signaling axis in macrophages, thus supporting their replication within the host.

## INTRODUCTION

Invasive cytosolic bacteria, including *Listeria*, *Shigella*, *Burkholderia*, *Francisella*, *Orientia*, and *Rickettsia* species, have developed strategies to induce their own uptake by phagocytosis and to circumvent host innate immune defenses for their intracellular survival (1, 2). Human infection with *Rickettsia* spp. occurs via infected hematophagous arthropods such as fleas, ticks, and human body lice (3), either through the bite or deposited as infected feces on skin and mucosal surfaces. Upon entry, *Rickettsia* spp. encounter tissue-resident immune cells, like macrophages (Mϕ). Activated Mϕ play a crucial role in either terminating an infection at an early stage, which commonly is the fate of nonpathogenic *Rickettsia* spp., or succumbing to bacterial replication and pathogen colonization as well as host dissemination to distant organs (3). After internalization into host cells, *Rickettsia* spp. escape from phagosomes and subvert host cytosolic defense mechanisms (i.e., autophagy and inflammasomes) to establish an intracytosolic replication niche. Recently, we reported that pathogenic *Rickettsia* spp. secret effectors to promote host colonization by modulating endoplasmic reticulum structures or by hijacking the autophagic defense pathway (4–9). Subversion of autolysosomal destruction to colonize the host cytosol exposes *Rickettsia* spp. to another cytosolic host sensor-regulated defense mechanism, the inflammasomes (1, 10). Inflammasomes are immune signaling complexes categorized into canonical (caspase-1 [Casp-1]) and noncanonical (murine Casp-11 or human Casp-4/5) inflammasomes. The inflammasome complex assembly involves the adaptor protein ASC and upstream sensors, including NLRP1, NLRP3, NLRC4, AIM2, and pyrin, which are primed by exogenous pathogen-associated molecular pattern molecules (PAMPs) and activated through endogenous damage-associated molecular pattern molecules (DAMPs). Initiation of the canonical inflammasome results in the activation of Casp-1. Active Casp-1 leads to the maturation of proinflammatory cytokines interleukin-1β (IL-1β) and IL-18 and the activation of gasdermin D (Gsdmd), the executor of pyroptosis (11, 12). Of note, recent findings have also suggested that active Casp-11 is capable of activating Gsdmd (11, 12). Although IL-1β is released by both canonical and noncanonical inflammasome pathways, IL-1α is preferentially released by the noncanonical inflammasome pathway (13–15). IL-1α is expressed by a wide range of hematopoietic and nonhematopoietic cell types, whereas IL-1β is primarily produced by myeloid cells (15). Importantly, although IL-1α and IL-1β signal through the same receptor, IL-1R, these two cytokines are not completely functionally redundant (15). Given the importance for both canonical and noncanonical inflammasome-mediated IL-1 signaling in limiting pathogen colonization, many bacteria have evolved strategies to block their activation (16–21). In fact, various pathogenic intracellular bacteria utilize their own effector repertoire to evade these pathways to successfully colonize and disseminate in their host cells (10, 22).

**FIG 1:**
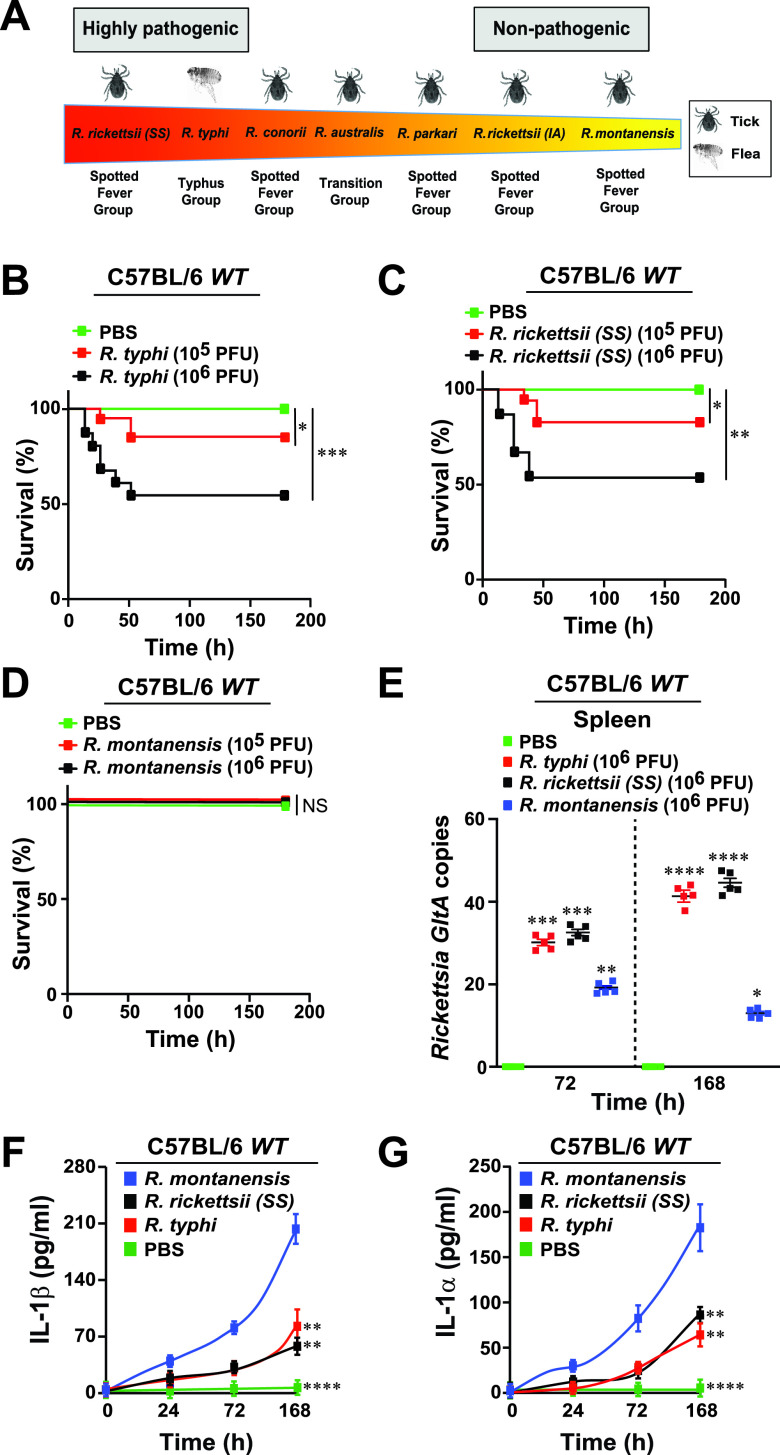
*In vivo* models of rickettsiosis. (A) *Rickettsia* spp. and their level of pathogenicity to humans (3, 44). (B to D) Establishment of a model of rickettsiosis (LD_25_ and LD_50_) for R. typhi (B), R. rickettsii (C) or R. montanensis (D) in C57BL/6J WT mice. Animals were injected via tail vein (i.v.) with different doses (10^5^ to 10^6^ PFU) of R. typhi, R. rickettsii, R. montanensis, or PBS (*n* = 12 for each treatment). Survival was monitored for 7 days. (E) Bacterial burden was tested in spleens of R. typhi-, R. rickettsii-, R. montanensis-, or PBS-injected WT mice (*n* = 5 per each treatment) shown in panels B to D by *GltA* RT-qPCR at days 3 and 7 (*n* = 5 for each treatment) using the host housekeeping gene *Gapdh* for normalization. (F and G) Serum samples from *Rickettsia*-infected mice described in panels B to D were analyzed for IL-1β (F) and IL-1α (G) production using the Legendplex kits (BioLegend), followed by flow cytometry analysis. Error bars in panels E to G represent means ± SEM from five independent experiments. NS, nonsignificant; *, *P ≤ *0.05; **, *P ≤ *0.01; ***, *P ≤ *0.005; ****, *P ≤ *0.001.

In the case of *Rickettsia*, our understanding of the role of inflammasomes in controlling host colonization is only now emerging (23–25). Specifically, how pathogenic *Rickettsia* spp. manipulate immune defenses to replicate within the host cytosol not only relies almost exclusively on data from tick-transmitted rickettsiae (e.g., members of the spotted fever group [SFG] or transition group [TRG]), but also shows that these pathogens likely employ species-specific strategies to evade host cytosolic defense mechanisms. For instance, Rickettsia australis, a pathogenic TRG member, benefited from ATG5-mediated autophagy induction and suppression of inflammasome-dependent IL-1β production to colonize both bone marrow-derived macrophages (BMDMs) (23) and mice (26). In contrast, Rickettsia parkeri, a mildly pathogenic member of SFG (see Fig. 1A below), utilizes its surface cell antigen Sca5 (OmpB) for protection against autophagic recognition and consequently benefits from inflammasome-mediated host cell death that antagonizes the action of type I interferon (IFN) in BMDMs and mice (24, 27). In contrast, our recent report on the flea-transmitted Rickettsia typhi (a pathogenic member of the typhus group [TG]), showed that R. typhi is ubiquitinated upon host entry and escapes autolysosomal fusion to establish an intracytosolic niche in nonphagocytic cells (9). These unexpected findings on how members of SFG, TRG, and TG *Rickettsia* differentially promote intracytosolic host survival prompted us to explore the underlying mechanism(s) by which pathogenic, but not nonpathogenic, *Rickettsia* spp. block immune defense responses to establish a replication niche in phagocytic cells, like Mϕ. Specifically, we sought to test the hypothesis that pathogenic, but not nonpathogenic, *Rickettsia* spp. reduce inflammasome-mediated IL-1 responses, thereby promoting their intracytosolic replication within host cells.

## RESULTS

### *In vivo* models of rickettsiosis.

At present, more than 30 *Rickettsia* spp. have been described globally, but less than a dozen is known to cause disease in humans, with some being notoriously pathogenic and associated with high morbidity and mortality, while others exert limited or no pathogenicity (Fig. 1A). We sought to test our hypothesis that pathogenic, but not nonpathogenic, *Rickettsia* spp. evade immune responses in host defense cells, like Mϕ, to replicate and disseminate. We simultaneously evaluated the cytosolic host defense responses between pathogenic (R. rickettsii strain Sheila Smith and R. typhi Wilmington) and nonpathogenic (Rickettsia montanensis) strains *in vivo*. We first established a mouse model of mild (approximate 25% lethal dose [∼LD_25_]) or more severe (∼LD_50_) rickettsiosis in C57BL/6J wild-type (WT) mice. For both R. rickettsii and R. typhi, LD_25_ or LD_50_ were achieved with doses of 10^5^ or 10^6^ PFU, respectively (Fig. 1B and C); however, R. montanensis-infected mice showed no signs of lethality at either 10^5^ or 10^6^ PFU (Fig. 1D). Bacterial burdens in spleens from infected C57BL/6J WT mice (only the dose of 10^6^ PFU is shown) confirmed successful infection with all three *Rickettsia* spp. at day 3 postinfection, while R. typhi- and R. rickettsii-infected WT mice displayed a significantly higher bacterial burden in the spleens compared to splenic tissues from R. montanensis-infected mice at day 7 (Fig. 1E). This correlated with the observed differences in the spleen sizes and weights of the infected animals (see Fig. S1 in the supplemental material). Given the earlier findings from other laboratories and ours (9, 23–28), we hypothesized that the observed dissimilarities in pathogenicity among *Rickettsia* spp. are likely linked to differences in host defense responses. Recent findings further suggest that the intracytosolic survival of different *Rickettsia* spp. is either supported or suppressed by immune defense responses (e.g., IFN-I, tumor necrosis factor alpha [TNF-α], or IL-1β) (23–28), thus leaving the precise mechanism to be determined. Therefore, we first sought to evaluate immune defense responses at the level of IL-1β and IL-1α cytokine secretion in the sera of R. typhi-, R. rickettsii-, and R. montanensis-infected animals. The increase in mortality and elevated bacterial burden correlated with reduced serum levels of both IL-1β and IL-1α cytokines (Fig. 1F and G), suggesting that reduced activation of both IL-1 signaling responses is a potential mechanism for lethality and survival of pathogenic *Rickettsia* spp.

10.1128/mBio.02918-21.1 FIG S1 Splenic data during infection of pathogenic and nonpathogenic *Rickettsia* species. (A) Representative images of spleens (at day 7) from C57BL/6J WT mice injected i.v. with R. typhi, R. rickettsii, R. montanensis, or PBS (dose of 10^6^ PFU). (B) Spleen weight from injected animals was evaluated at day 7 day (*n* = 5). Error bars in panel B represent means ± SEM from five independent experiments. *, *P ≤ *0.05; ***, *P ≤ *0.005. Download FIG S1, EPS file, 1.7 MB. Copyright © 2022 Voss et al. 2022 Voss et al. https://creativecommons.org/licenses/by/4.0/ This content is distributed under the terms of the Creative Commons Attribution 4.0 International license.

### Antirickettsial activity of IL-1α is involved in restricting *Rickettsia* infection.

**FIG 2:**
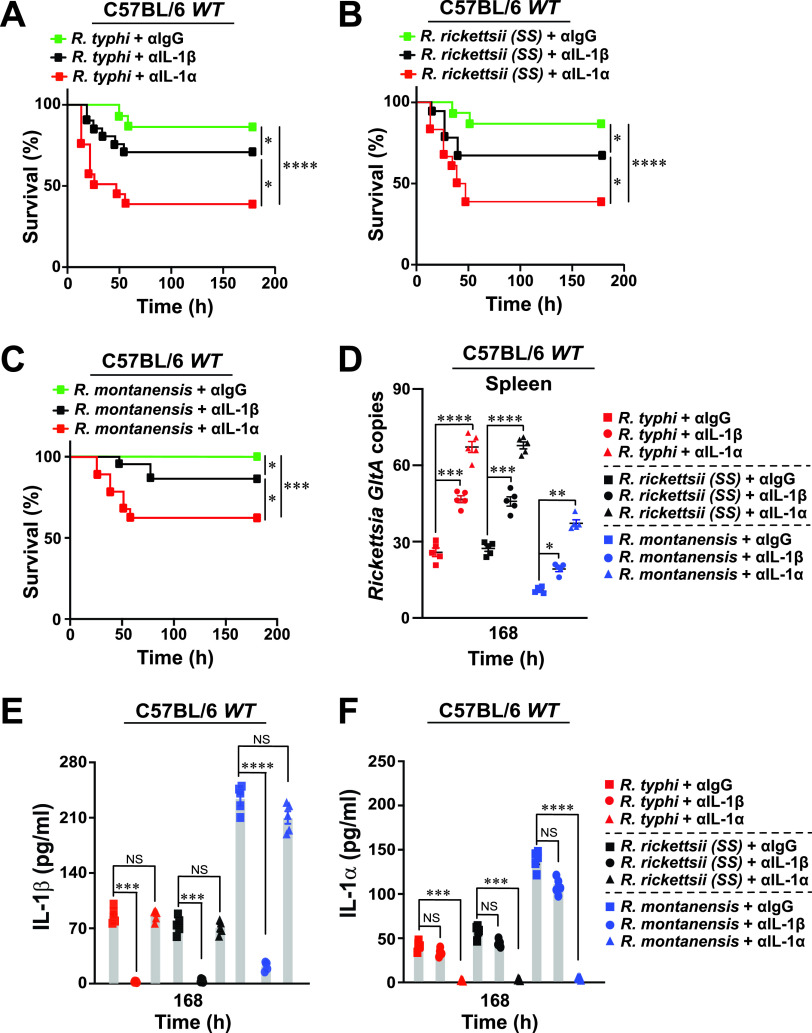
Neutralization of IL-1α activity augments mortality of pathogenic and nonpathogenic *Rickettsia*-induced rickettsiosis. (A to C) C57BL/6J WT mice were injected via tail vein (i.v.) with 10^5^ PFU of R. typhi (A), R. rickettsii (B), or R. montanensis (C) (A to C; *n* = 12 for each treatment), followed by a subsequent injection (i.v.) with anti-IL-1β, anti-IL-1α, or anti-IgG isotype control antibody (Ab) (250 μg Ab/mouse). Survival was monitored for 7 days. (D) Bacterial burden was tested in the spleens of the Ab-treated R. typhi-, R. rickettsii-, and R. montanensis-injected WT mice shown in panels A to C by *GltA* RT-qPCR at day 7 (*n* = 5 for each treatment) using the host housekeeping gene *Gapdh* for normalization. (E and F) Serum samples from mice described in panels A to C were analyzed for IL-1β (E) and IL-1α (F) production at day 7 (*n* = 5 for each treatment) using the Legendplex kits (BioLegend), followed by flow cytometry analysis. Error bars in panels D to F represent means ± SEM from five independent experiments. NS, nonsignificant; *, *P ≤ *0.05; **, *P ≤ *0.01; ***, *P ≤ *0.005; ****, *P ≤ *0.001.

**FIG 3:**
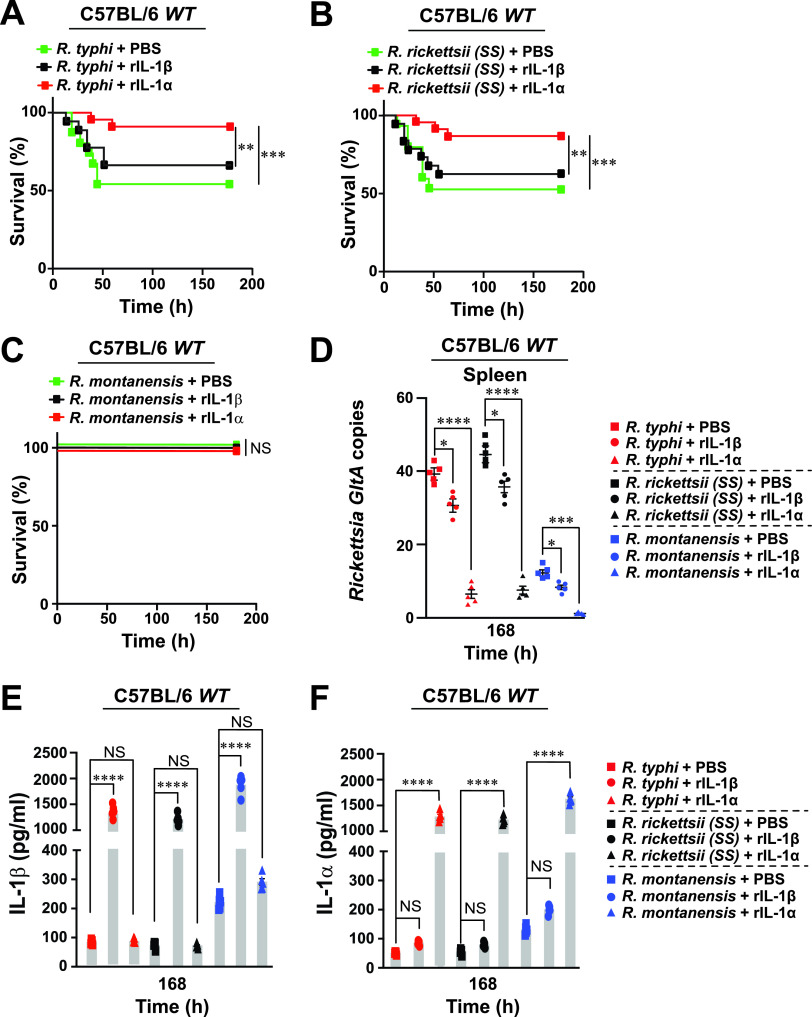
Administration of recombinant IL-1α rescues fatal *Rickettsia*-induced rickettsiosis. (A to C) C57BL/6J WT mice were injected i.v. with rIL-1β or rIL-1α protein (500 ng/mouse), followed by infection (24 h post-protein injection) with 10^6^ PFU of R. typhi (A), R. rickettsii (B), R. montanensis (C), or PBS (A to C; *n* = 15 for each treatment). Survival was monitored for 7 days. (D) Bacterial burden was tested in the spleens of the protein-treated R. typhi-, R. rickettsii-, R. montanensis-, or PBS-injected WT mice shown in panels A to C by *GltA* RT-qPCR at day 7 (*n* = 5 for each treatment), using the housekeeping gene *Gapdh* for normalization. (E and F) Serum samples from the mice described in panels A to C were analyzed for IL-1β (E) and IL-1α (F) production at day 7 (*n* = 5 for each treatment) using the Legendplex kits (BioLegend), followed by flow cytometry analysis. Error bars in panels D to F represent means ± SEM from five independent experiments. NS, nonsignificant; *, *P ≤ *0.05; **, *P ≤ *0.01; ***, *P ≤ *0.005; ****, *P ≤ *0.001.

To characterize further the role of IL-1α and IL-1β cytokines in restricting nonpathogenic and pathogenic *Rickettsia* spp. *in vivo*, IL-1α or IL-1β function was neutralized via tail vein (intravenous [i.v.]) injection with anti-IL-1α, anti-IL-1β, or anti-IgG-isotype control antibodies (Abs) into C57BL/6J WT mice in our established model of mild rickettsiosis (LD_25_; ∼10^5^ PFU) (Fig. 1B to D). Neutralization of IL-1α, and to a much lesser extent IL-1β, was associated with a significant increase in the mortality of R. typhi-, R. rickettsii-, and R. montanensis-infected mice (Fig. 2A to C) and resulted in the development of splenomegaly (see Fig. S2 in the supplemental material), which correlated with an increase in bacterial loads in the spleen (Fig. 2D). The efficiency of Ab-mediated blocking was confirmed by measuring the levels of IL-1β and IL-1α cytokine in the sera of the rickettsia-infected mice (Fig. 2E and F). Next, we sought to determine the effect of administering recombinant IL-1α (rIL-1α) or rIL-1β proteins on *Rickettsia* colonization *in vivo*. Accordingly, we administered (i.v.) endotoxin-free rIL-1α and rIL-1β proteins following infection with R. typhi, R. rickettsii, and R. montanensis. Pretreatment of mice with rIL-1α and, to a lesser extent, rIL-1β protected C57BL/6J WT mice from pathogenic *Rickettsia*-induced lethality (Fig. 3A to C), with both reduced splenomegaly (see Fig. S3 in the supplemental material) and decreased splenic bacterial burdens (Fig. 3D). Moreover, the observed phenotypes correlated with the measured IL-1α and IL-1β serum concentrations (Fig. 3E and F). Collectively, these findings suggest that IL-1α and, to a lesser extent, IL-1β are involved in restricting the replication and colonization of nonpathogenic and pathogenic *Rickettsia* spp. in C57BL/6J WT mice.

10.1128/mBio.02918-21.2 FIG S2 Splenic data after IL-1 signaling neutralization during infection of pathogenic and nonpathogenic *Rickettsia* species. (A) Representative images of spleens (day 7) from C57BL/6J WT mice injected i.v. with R. typhi, R. rickettsii, or R. montanensis (dose of 10^5^ PFU), followed by administration of anti-IL-1β, anti-IL-1α, or IgG isotype control antibody (Ab) (250 μg Ab/mouse) (24 h post-Ab injection). (B) Spleen weight from injected animals was evaluated at day 7 (*n* = 5). Error bars in panel B represent means ± SEM from five independent experiments. *, *P ≤ *0.05; **, *P ≤ *0.01; ***, *P ≤ *0.005. Download FIG S2, EPS file, 3.2 MB. Copyright © 2022 Voss et al. 2022 Voss et al. https://creativecommons.org/licenses/by/4.0/ This content is distributed under the terms of the Creative Commons Attribution 4.0 International license.

10.1128/mBio.02918-21.3 FIG S3 Splenic data after administration of recombinant IL-1α and IL-1β proteins during infection of pathogenic and nonpathogenic *Rickettsia* species. (A) Representative images of spleens (day 7) from C57BL/6J WT mice injected i.v. with rIL-1β or rIL-1α protein, followed by administration of R. typhi, R. rickettsii, R. montanensis, or PBS (dose of 10^6^ PFU) at day 7. (B) Spleen weight from injected animals was evaluated at day 7 day (*n* = 5). Error bars in panel B represent means ± SEM from five independent experiments. *, *P ≤ *0.05; **, *P ≤ *0.01; ***, *P ≤ *0.005. Download FIG S3, EPS file, 4.0 MB. Copyright © 2022 Voss et al. 2022 Voss et al. https://creativecommons.org/licenses/by/4.0/ This content is distributed under the terms of the Creative Commons Attribution 4.0 International license.

### Pathogenic, but not nonpathogenic, *Rickettsia* species block IL-1α secretion and avoid pro-IL-1α induction to establish a replication niche in macrophages.

**FIG 4:**
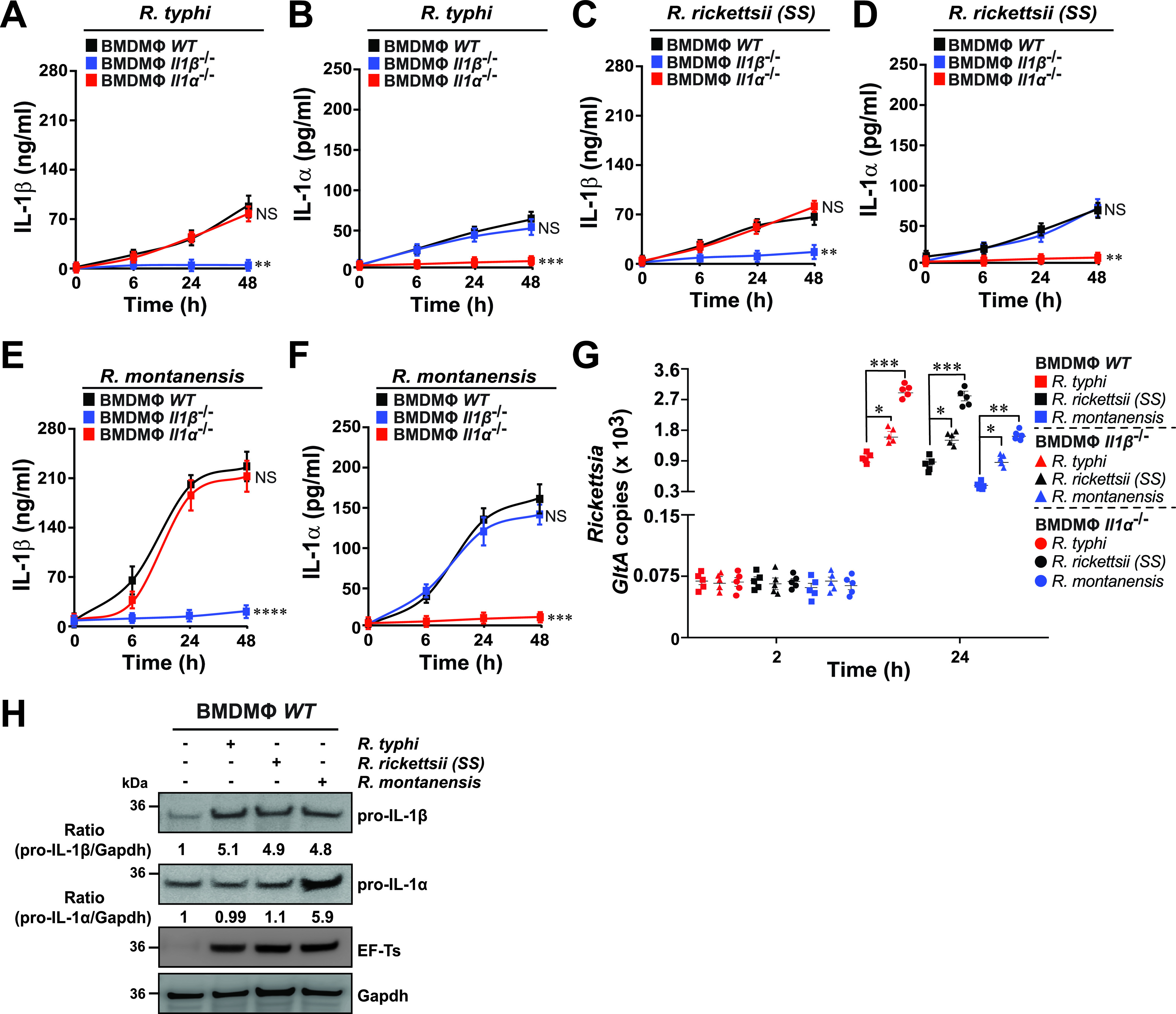
IL-1α, but not IL-1β, contributes to the survival of *Rickettsia* species in macrophages. (A to F) BMDMs from WT, *Il-1β^−/−^*, or *Il-1α^−/−^* mice were infected with R. typhi, R. rickettsii, or R. montanensis (MOI = 50) for 0, 6, 24, and 48 h. Culture supernatants were analyzed for production of IL-1β (A, C, and E) and IL-1α (B, D, and F) using Legendplex kits (BioLegend), followed by flow cytometry. (G) Bacterial burden in *Rickettsia*-infected BMDMs from WT, *Il-1β^−/−^*, or *Il-1α^−/−^* mice was evaluated at 2 and 24 h postinfection by *GltA* RT-qPCR. Expression of the housekeeping gene *Gapdh* was used for normalization. (H) BMDMs from WT mice were either left uninfected (−) or were infected with R. typhi, R. rickettsii, or R. montanensis (MOI = 50) for 24 h. Lysates were immunoblotted with anti-IL-1α, anti-IL-1β, anti-ET-Ts, and anti-Gapdh Abs. Densitometry was performed using Fiji software, and data representing the fold change ratios of pro-IL-1β/Gapdh or pro-IL-1α/Gapdh between uninfected and infected cells are shown. Immunoblot data are representative of three independent experiments. Error bars in panels A to G represent means ± SEM from five independent experiments. NS, nonsignificant; *, *P ≤ *0.05; **, *P ≤ *0.01; ***, *P ≤ *0.005; ****, *P ≤ *0.001.

As Mϕ are one of the cell types first encountered during infection by *Rickettsia* spp. and are considered to play a crucial role in either terminating the infection early at the skin site or allow initial pathogen colonization and subsequent dissemination within the infected host (3), we tested the hypothesis that pathogenic, but not nonpathogenic, rickettsiae suppress Mϕ immune responses. In this effort, we determine the importance of IL-1α and IL-1β in restricting the replication of *Rickettsia* spp. by infecting BMDMs derived from WT, *Il-1β^−/−^*, or *Il-1α^−/−^* mice with pathogenic R. typhi and R. rickettsii or nonpathogenic R. montanensis and assessed the cytokine levels of IL-1β and IL-1α in cultured supernatants as well as bacterial burdens. Infection of *Il-1β^−/−^* or *Il-1α^−/−^* BMDMs with *Rickettsia* spp. did not result in the secretion of either IL-1β or IL-1α, respectively, compared to infected WT BMDMs (Fig. 4A to F). Moreover, infections with R. montanensis resulted in overall higher IL-1 responses and lower bacterial burden compared to infections performed with R. typhi or R. rickettsii in WT, *Il-1β^−/−^*, or *Il-1α^−/−^* BMDMs (Fig. 4A to G). Of note, the bacterial burden of all three *Rickettsia* spp. in infected *Il-1α^−/−^* BMDMs was higher than the levels detected in *Il-1β^−/−^* or WT BMDMs (Fig. 4G), suggesting that IL-1α and, to significantly lesser extent, IL-1β play a role in restricting rickettsia survival. In agreement with our *in vivo* data, infection assays using BMDMs also displayed an overall higher bacterial burden of both pathogenic *Rickettsia* spp., compared to the nonpathogenic *Rickettsia* strain (Fig. 1E and Fig. 4G). We further tested the protein expression levels of pro-IL-1β and pro-IL-1α upon bacterial-infection of WT BMDMs and showed that pro-IL-1β levels were induced by all three *Rickettsia* spp. to similar levels (∼5-fold) compared to uninfected WT BMDMs (Fig. 4H). Intriguingly, only R. montanensis-infected WT BMDMs produced significantly higher levels of pro-IL-1α than R. typhi- or R. rickettsii-infected Mϕ (Fig. 4H).

### Intracytosolic replication of pathogenic *Rickettsia* species in macrophages depends on the inhibition of IL-1 cytokine secretion via a caspase-11–Gsdmd-dependent pathway.

**FIG 5:**
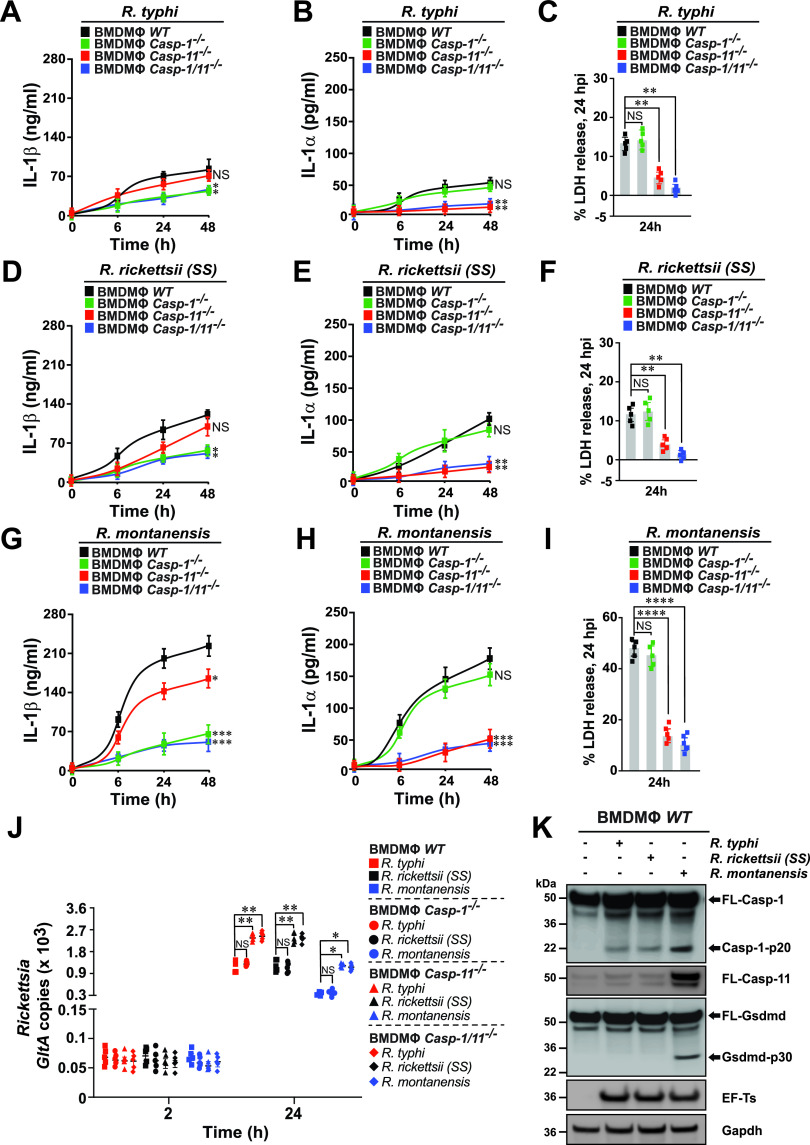
Pathogenic, but not nonpathogenic, *Rickettsia* spp. limit caspase-1- and caspase-11-dependent IL-1 signaling to facilitate their intracellular replication in macrophages. (A to I) BMDMs from WT, *Casp-1^−/−^*, *Casp-11^−/−^*, or *Casp-1/11^−/−^* mice were infected with R. typhi (A to C), R. rickettsii (D to F), or R. montanensis (G to I) (MOI = 50) for 0, 6, 24, and 48 h. Culture supernatants were analyzed for production of IL-1β (A, D, and G) and IL-1α (B, E, and H) using Legendplex kits (BioLegend), followed by flow cytometry. BMDM cell death at 24 h postinfection was measured by lactate dehydrogenase (LDH) release assay (C, F, and I). (J) Bacterial burdens in infected BMDMs were evaluated 2 and 24 h postinfection by *GltA* RT-qPCR. Expression of the host housekeeping gene *Gapdh* was used for normalization. (K) Western analysis of Casp-1, Casp-11, and Gsdmd induction and processing at 24 h postinfection with R. typhi, R. rickettsii, or R. montanensis using anti-Casp-1, anti-Casp-11, and anti-Gsdmd Abs. Reblotting with *Rickettsia*-specific anti-EF-Ts and host-cell-specific anti-Gapdh Abs served as infection and equal loading controls, respectively. Densitometry was performed using Fiji software, and data representing the fold change ratios of Casp-1-p20/FL-Casp-1, Gsdmd-p30/FL-Gsdmd, or Casp-11/Gapdh between uninfected and infected cells are shown. Error bars in panels A to J represent means ± SEM from five independent experiments. NS, nonsignificant; *, *P ≤ *0.05; **, *P ≤ *0.01; ***, *P ≤ *0.005; ****, *P ≤ *0.001. Immunoblot data are representative of three independent experiments.

As our findings suggest that pathogenic, but not nonpathogenic, *Rickettsia* spp. prevent the activation of signaling pathways required for IL-1α and IL-1β production and release, we explored the mechanism of IL-1 signaling in greater detail. As IL-1 signaling responses commonly involve the canonical and noncanonical inflammasome pathways, which in turn involves the proteolytic processing of both cytokines by activated caspase-1 (Casp-1 [canonical]) and Casp-11 [noncanonical]), respectively, we assessed their potential role in regulating replication of nonpathogenic versus pathogenic *Rickettsia* in BMDMs. In this effort, BMDMs derived from WT, *Casp-1^−/−^*, *Casp-11^−/−^*, and *Casp-1/11^−/−^* mice were infected with R. typhi, R. rickettsii, and R. montanensis, and the levels of IL-1β and IL-1α and cell death, as well as bacterial burdens, were evaluated over the course of infection. Our assays revealed that Casp-11 was involved in the secretion of cleaved IL-1α upon infection of BMDMs with nonpathogenic and pathogenic *Rickettsia* spp. (Fig. 5A, B, D, E, G, and H). In addition, Casp-11 deficiency resulted in a significant decrease in host cell death (Fig. 5C, F, and I). Casp-1 deficiency (*Casp-1^−/−^*) caused a significant decrease in IL-1β production (Fig. 5A, D, and G, green lines), while IL-1α secretion as well as the level of cell death remained unaffected during infection with all three *Rickettsia* spp. (Fig. 5B, C, E, F, H, and I, green lines and symbols). Analysis of bacterial burdens further revealed a prominent role for Casp-11, but not for Casp-1, in restricting the replication of both pathogenic and nonpathogenic *Rickettsia* spp. (Fig. 5J; WT [squares], *Casp-1^−/−^* [circles], *Casp-11*^−/−^ [triangles], and *Casp-1*/*11^−/−^* [diamonds]). As our findings indicate that infections with R. typhi and R. rickettsii resulted in a significant reduction of IL-1α secretion, likely via a Casp-11-dependent mechanism, we assessed the expression and activation status of Casp-1 and Casp-11 via Western blot analyses. R. montanensis infection resulted in a robust activation of Casp-1, as indicative of the detection of the Casp-1–p20 fragment (Fig. 5K). In contrast, infection with R. typhi and R. rickettsii spp. resulted in a lower activation of Casp-1 (∼5-fold) (Fig. 5K). Intriguingly, only infection with R. montanensis resulted in a robust induction of Casp-11 (∼8-fold) compared to infection data using both pathogenic *Rickettsia* spp. (Fig. 5K). To test if IL-1 cytokine secretion is dependent on the bacterial load, we heat inactivated both pathogenic and nonpathogenic *Rickettsia* spp. and showed that IL-1β and IL-1α release was significantly impaired compared to that in infections using viable *Rickettsia* spp., a phenotype more strongly observed in infections using R. montanensis (see Fig. S4 in the supplemental material). As IL-1 cytokine secretion is dependent on Gsdmd, the pore-forming executor of pyroptosis (29–32), we assessed the proteolytic processing of Gsdmd and showed that only R. montanensis infection resulted in a robust cleavage of Gsdmd (∼8-fold), as indicative by the detection of the Gsdmd-p30 fragment (Fig. 5K). In support of our findings, we showed that infection of *Gsdmd^−/−^* BMDMs with nonpathogenic and pathogenic *Rickettsia* spp. released significantly lower levels of IL-1β and IL-1α than infection of WT BMDMs (see Fig. S5A and B in the supplemental material). Furthermore, analysis of bacterial burdens provided additional evidence that Gsdmd plays a role in restricting the replication of R. typhi, R. rickettsii, and R. montanensis (Fig. S5C). These findings suggest that pathogenic, unlike nonpathogenic, *Rickettsia* spp. suppress IL-1 cytokine secretion via a Casp-11–Gsdmd-dependent pathway to support an intracytosolic replication in Mϕ.

10.1128/mBio.02918-21.4 FIG S4 Heat inactivation of *Rickettsia* species resulted in a reduced IL-1α and IL-1β cytokine release by macrophages. (A and B) BMDMs from WT mice were infected with heat-inactivated or non-heat-treated R. typhi, R. rickettsii, or R. montanensis (MOI = 50) for 24 h. Culture supernatants were analyzed for production of IL-1β (A) and IL-1α (B) using Legendplex kits (BioLegend), followed by flow cytometry. (C) Bacterial burden in *Rickettsia*-infected BMDMs from WT mice was evaluated at 2 and 24 h postinfection by *GltA* RT-qPCR. Expression of the housekeeping gene *Gapdh* was used for normalization. Error bars in panels A to C represent means ± SEM from five independent experiments. NS, nonsignificant; **, *P ≤ *0.01; ***, *P ≤ *0.005; ****, *P ≤ *0.001. Download FIG S4, EPS file, 1.7 MB. Copyright © 2022 Voss et al. 2022 Voss et al. https://creativecommons.org/licenses/by/4.0/ This content is distributed under the terms of the Creative Commons Attribution 4.0 International license.

10.1128/mBio.02918-21.5 FIG S5 Gsdmd is involved in the release of IL-1α and IL-1β by macrophages upon *Rickettsia* infection. (A and B) BMDMs from WT and *Gsdmd^−/−^* mice were infected with R. typhi, R. rickettsii, or R. montanensis (MOI = 50) for 24 h. Culture supernatants were analyzed for production of IL-1β (A) and IL-1α (B) using Legendplex kits (BioLegend), followed by flow cytometry. (C) Bacterial burden in *Rickettsia*-infected BMDMs from WT and *Gsdmd^−/−^* mice was evaluated at 2 and 24 h postinfection by *GltA* RT-qPCR. Expression of the housekeeping gene *Gapdh* was used for normalization. Error bars in panels A to C represent means ± SEM from five independent experiments. NS, nonsignificant; *, *P ≤ *0.05; **, *P ≤ *0.01; ***, *P ≤ *0.005. Download FIG S5, EPS file, 1.7 MB. Copyright © 2022 Voss et al. 2022 Voss et al. https://creativecommons.org/licenses/by/4.0/ This content is distributed under the terms of the Creative Commons Attribution 4.0 International license.

### Secretion of IL-1α by macrophages is crucial in restricting the replication of pathogenic and nonpathogenic *Rickettsia* species *in vivo*.

**FIG 6:**
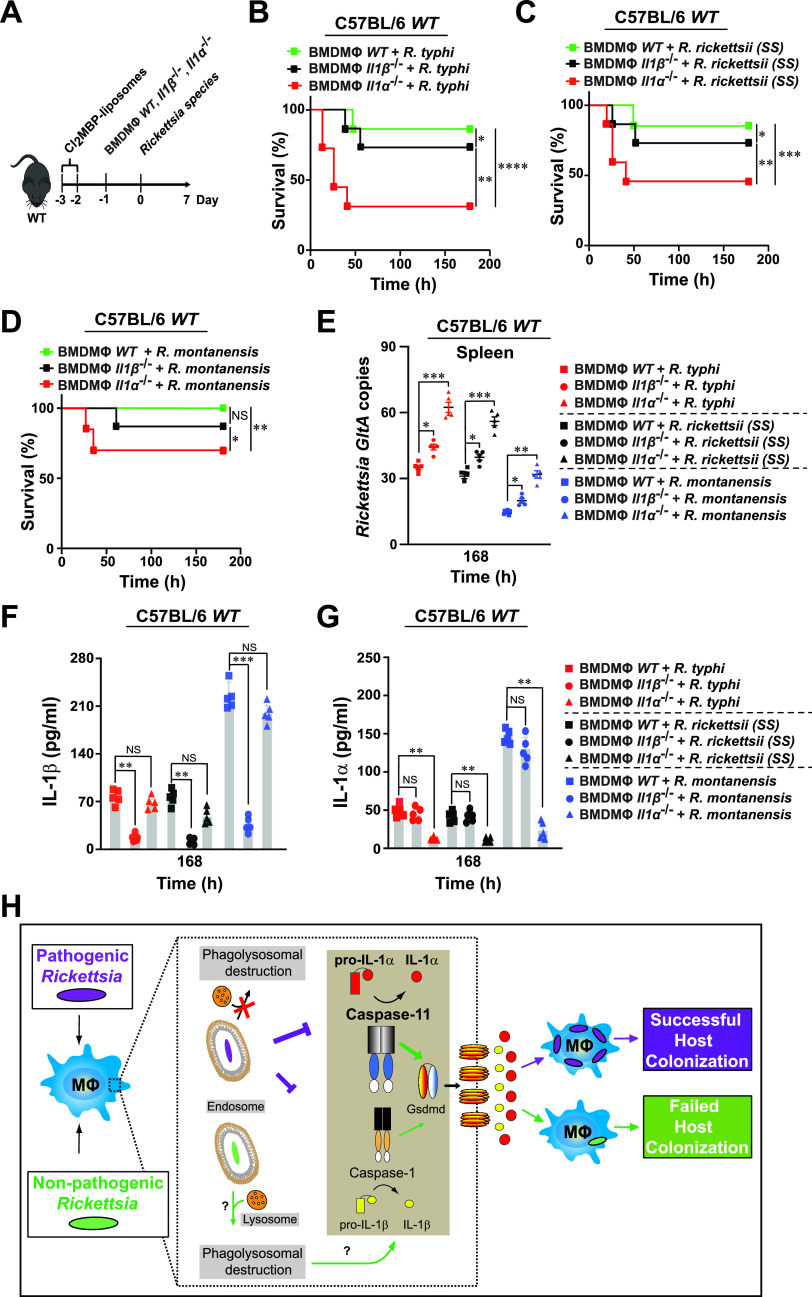
Macrophage-dependent secretion of IL-1α contributes more than IL-1β for controlling the survival and colonization of pathogenic and nonpathogenic *Rickettsia* species. (A to D) Dichloromethylene biphosphate (Cl_2_MBP)-treated C57BL/6J WT mice were injected (i.v.) with WT, *Il-1β^−/−^*, or *Il-1α^−/−^* BMDMs (5 × 10^6^ cells/mouse), followed by infection (24 h post-Mϕ transfer) with R. typhi (B), R. rickettsii (C), or R. montanensis (D) (dose 10^5^ PFU) (B to D; *n* = 12 for each treatment). Survival was monitored for 7 days. (E) Bacterial burden was tested in spleens from mice described in panels B to D by *GltA* RT-qPCR at day 7 (*n* = 5 for each treatment), using the housekeeping gene *Gapdh* for normalization. (F to G) Serum samples from mice described in panels B to D were analyzed for IL-1β (F) and IL-1α (G) production at day 7 (*n* = 5 for each treatment) using the Legendplex kits (BioLegend), followed by flow cytometry analysis. Error bars in panels E to G represent means ± SEM from five independent experiments. NS, nonsignificant; *, *P ≤ *0.05; **, *P ≤ *0.01; ***, *P ≤ *0.005; ****, *P ≤ *0.001. (H) Proposed working model on how pathogenic *Rickettsia* spp. suppress Casp-1- and Casp-11-dependent IL-1 signaling responses to establish a replication niche *in vitro* and *in vivo*. Of note, the majority of nonpathogenic *Rickettsia* spp. are likely destroyed by phagolysosomal fusion, while a subpopulation may escape lysosomal fusion, ultimately allowing for the induction of inflammasome-mediated IL-1 responses.

To examine whether secretion of IL-1 by Mϕ limits the replication of *Rickettsia* spp. *in vivo*, we first injected (i.v.) WT mice with phosphate-buffered saline (PBS)- or dichloromethylene biphosphate (Cl_2_MBP)-liposomes to deplete endogenous macrophages as described previously (33). Next, PBS- or Cl_2_MBP-liposome-treated WT mice were injected (i.v.) with BMDMs isolated from WT, *Il-1β^−/−^*, or *Il-1α^−/−^* mice prior to infection with R. typhi, R. rickettsii, or R. montanensis. Strikingly, adoptive transfer of *Il-1α^−/−^* BMDMs, but not *Il-1β^−/−^* or WT Mϕ, significantly increased the mortality of Cl_2_MBP- but not PBS-treated WT mice injected with all three *Rickettsia* spp., reaching levels similar to the survival percentages observed in IL-1α Ab neutralization studies (Fig. 2 and Fig. 6A to D; see Fig. S6 in the supplemental material). Moreover, transfer of *Il-1α^−/−^* BMDMs resulted in the development of splenomegaly (see Fig. S7 in the supplemental material) and an increase in bacterial burden in the spleens of Cl_2_MBP-treated WT mice (Fig. 6E), which correlated with a decrease in IL-1α serum concentrations without affecting IL-1β serum levels (Fig. 6F and G). In contrast, transfer of *Il-1β^−/−^* BMDMs had an overall lesser effect on the severity of rickettsiosis, as evidenced by a lower mortality rate, smaller spleen size, and lower bacterial burden in Cl_2_MBP-treated WT mice (Fig. 6A to E; see Fig. S7 in the supplemental material), which is in agreement with our IL-1β Ab neutralization data (Fig. 2). In addition, transfer of *Il-1β^−/−^* BMDMs resulted in a decrease in IL-1β serum concentrations without affecting IL-1α serum levels (Fig. 6F and G). Collectively, these data suggest that modulation of expression and secretion of IL-1α by macrophages is important to limit the replication of *Rickettsia* spp. *in vivo*.

10.1128/mBio.02918-21.6 FIG S6 Survival data after adoptive transfer of WT, *Il-1β^−/−^*, or *Il-1α^−/−^* macrophages and infection of pathogenic and nonpathogenic *Rickettsia* species into PBS-liposome-treated C57BL/6J WT mice. (A to D) PBS-treated C57BL/6J WT mice were injected i.v. with WT, *Il-1β^−/−^*, or *Il-1α^−/−^* BMDMs (5 × 10^6^ cells/mouse), followed by infection (24 h post-Mϕ transfer) with R. typhi (B), R. rickettsii (C), R. montanensis (D), or PBS (dose of 10^5^ PFU) (B to D; *n* = 12 for each treatment). Survival was monitored for 7 days. Download FIG S6, EPS file, 1.6 MB. Copyright © 2022 Voss et al. 2022 Voss et al. https://creativecommons.org/licenses/by/4.0/ This content is distributed under the terms of the Creative Commons Attribution 4.0 International license.

10.1128/mBio.02918-21.7 FIG S7 Splenic data after adoptive transfer of WT, *Il-1β^−/−^*, or *Il-1α^−/−^* macrophages and infection of pathogenic and nonpathogenic *Rickettsia* species into dichloromethylene biphosphate (Cl_2_MBP)-treated C57BL/6J WT mice. (A) Representative images of spleens (day 7) from Cl_2_MBP-treated C57BL/6J WT mice injected i.v. with WT, *Il-1β^−/−^*, or *Il-1α^−/−^* BMDMs (5 × 10^6^ cells/mouse), followed by administration (24 h post-Mϕ transfer) of R. typhi, R. rickettsii, R. montanensis, or PBS (dose of 10^5^ PFU). (B) Spleen weight from injected animals was evaluated at day 7 (*n* = 5). Error bars in panels B represent means ± SEM from five independent experiments. NS, nonsignificant; *, *P ≤ *0.05; **, *P ≤ *0.01. Download FIG S7, EPS file, 3.8 MB. Copyright © 2022 Voss et al. 2022 Voss et al. https://creativecommons.org/licenses/by/4.0/ This content is distributed under the terms of the Creative Commons Attribution 4.0 International license.

## DISCUSSION

Obligate intracellular bacterial pathogens, which successfully reside and replicate within the host cell, overcome responses of innate immune defense surveillance (e.g., inflammasomes and autophagy). However, in the case of strict obligate intracytosolic *Rickettsia* spp., the roles for both inflammasome and autophagy to restrict their replication in endothelial cells and immune cells, like Mϕ, is only now emerging, although without consistent mechanistical insights (23–28, 34). Given these knowledge gaps and our current lack of understanding on how TG *Rickettsia* spp. evade immune defense responses to facilitate host colonization, we established an animal model of rickettsiosis using C57BL/6J WT mice by comparing development of both mild (∼LD_25_) and more severe (∼LD_50_) disease for two pathogenic spp., R. rickettsii and R. typhi. In fact, data of disease severity correlated with the bacterial burdens detected in the mice spleens. We also observed that infections with pathogenic, but not nonpathogenic, *Rickettsia* spp. resulted in a reduced serum response of both proinflammatory cytokines, IL-1β and IL-1α, which is likely the result of pathogenic *Rickettsia* spp. to evade canonical and noncanonical inflammasome-dependent defense sensing, of which the former is in agreement with recent studies using *R. australis* (26). Thus, our data suggest that inhibition of both canonical and noncanonical inflammasome-dependent signaling contributes to the enhanced survival and colonization of pathogenic *Rickettsia* spp. in our *in vivo* experiments.

Our data further suggest that IL-1α and, to a significantly lesser extent, IL-1β play a role in limiting rickettsial infection *in vivo*. By testing the putative antirickettsial capabilities of these cytokines through employing Ab neutralization and recombinant protein assays, we showed that IL-1α and, to a much lesser extent, IL-1β was able to restrict the replication and colonization of both nonpathogenic and pathogenic *Rickettsia* spp. *in vivo*. Our current understanding by which *Rickettsia* spp. evade host-induced antibacterial activities, and in particular, how cytosolic rickettsiae overcome host immune surveillance in defense cells like Mϕ, is primarily based on reports that are not aligned with one another (23–25, 27). Thus, we sought to address the hypothesis that pathogenic R. typhi and R. rickettsii spp., but not the nonpathogenic species R. montanensis, evade innate immune defense responses in order to establish an intracytosolic replication niche in Mϕ. In agreement with our *in vivo* infection models, R. montanensis-infected WT BMDMs produced higher levels of IL-1α and IL-1β cytokines and displayed reduced bacterial loads during the course of infection, compared to R. typhi- or R. rickettsii-infected BMDMs. These data support the notion that nonpathogenic, but not pathogenic, *Rickettsia* spp. are more efficiently cleared by Mϕ, a mechanism that further supports the previously published findings with SFG *Rickettsia* using THP-1 cells, a human macrophage-like cell line (35). Collectively, our presented data strengthen our hypothesis that pathogenic, but not nonpathogenic, *Rickettsia* spp. suppress antirickettsial inflammasome-dependent IL-1 cytokine responses to establish an intracytosolic replication niche in Mϕ.

Given that IL-1 signaling is modulated through inflammasome-dependent Casp-1, Casp-11, and Gsdmd activation, we tested the role of both caspases as well as Gsdmd, and found that nonpathogenic, but not pathogenic, *Rickettsia* spp. ensured Casp-1 activation and Casp-11 induction, which ultimately resulted in the proteolytic processing of Gsdmd and release of IL-1α and IL-1β cytokines. Intriguingly, the lack of Casp-11 induction by pathogenic, but not nonpathogenic, *Rickettsia* spp. suggests that the membrane-bound lipopolysaccharide (LPS) of R. typhi and R. rickettsii spp. is likely less immunogenic than that of R. montanensis, which is further supported by our recent reports (36, 37). Our findings further suggest that pathogenic, compared to nonpathogenic, *Rickettsia* spp. benefit from evasion of the Casp-11–Gsdmd–IL-1α signaling axis to establish a replication niche in Mϕ, as evidenced by the increased replication in Mϕ from *Casp-11^−/−^*, *Casp-1/11^−/−^*, or *Gsdmd^−/−^* mice compared to WT and *Casp-1^−/−^* BMDMs. Finally, we sought to determine the role of IL-1 cytokine responses by Mϕ in restricting the replication of *Rickettsia* spp. and showed that transfer of *Il-1α^−/−^* BMDMs and, to much lesser extent, the administration of *Il-1β^−/−^* BMDMs exacerbated the disease progression in WT mice injected with either pathogenic or nonpathogenic *Rickettsia* spp. It is worth noting that the observed differences in IL-1α release could be partially attributed to alternative mechanisms, including the retainment of IL-1α in the cytosol or nucleus, the dependence of IL-1β, and/or the association with the decoy receptor of IL-1 (IL-1R2), and future experiments are under way to address these possibilities (15, 29, 33, 38–40). Also, IL-1α is produced by other immune cells, such as neutrophils (15). Although our study did not evaluate a potential contributing role of neutrophils, preceding findings suggest that neutrophils did not alter the course of rickettsiosis or contribute to the restriction of bacterial growth (28).

Importantly, preceding findings suggest that intracellular pathogens, like rickettsiae, not only encounter inflammasome-dependent defense mechanisms but also are confronted by another cytosolic defense pathway, autophagy (23, 25). Both responses not only are key to mount the appropriate host defense responses (16, 18), but also are functionally interconnected. In fact, recent reports indicated that autophagy acts on intracellular microbes upstream of the inflammasome and thereby functions as a negative regulator by degrading inflammasome components (10, 16, 18, 22). In the case of rickettsiae, however, the role of autophagy in regulating inflammasome responses to facilitate their host colonization remains inconclusive. For instance, *R. australis*, a pathogenic TRG member, benefited from ATG5-dependent autophagy induction and suppression of inflammasome-dependent IL-1β production to colonize Mϕ (23, 25). In contrast, *R. parkeri*, a mildly pathogenic member of SFG, demonstrated that its surface protein OmpB is critical for protecting against autophagic recognition, while evasion of autophagy was critical for invasion of BMDMs and WT mice by *R. parkeri* (24, 27). Intriguingly, our recent report on R. typhi showed that R. typhi is ubiquitinated upon host entry, induces autophagy, but escapes autophagolysosomal maturation for intracellular colonization in nonphagocytic cells (9). Given these reports by others and our recent findings, it is tempting to speculate that pathogenic, but not nonpathogenic, *Rickettsia* spp. induce autophagy to downregulate inflammasome-dependent IL-1β and IL-1α cytokine responses to establish an intracytosolic replication niche *in vitro* and *in vivo*, and our future research will address this possibility.

Overall, our findings present a previously unappreciated model of host invasion by which pathogenic, but not nonpathogenic, *Rickettsia* spp. avoid the activation of signaling pathways required for IL-1α production and release—likely via the suppression of the Casp-11–Gsdmd signaling pathway—to facilitate their intracytosolic replication in Mϕ and ultimately cause host colonization (Fig. 6H).

## MATERIALS AND METHODS

### Animals.

All experiments were conducted in fully AAALAC-accredited program using 8- to 10-week-old female C57BL/6J WT mice in a specific-pathogen-free environment according to the University of Maryland School of Medicine Institutional Animal Care and Use Committee (IACUC) in compliance with the National Institutes of Health guide (41).

### Antibodies and reagents.

Anti-IL-1α (clone ALF-161), anti-IL-1β (clone B122), and an isotype control IgG (Armenian hamster IgG) antibody (Ab) were purchased from BioXCell. Anticaspase (anti-Casp-1) Ab was purchased from Adipogen, while anti-Casp-11 (clone EPR18628) and anti-Gsdmd (clone EPR19828) Abs were obtained from Abcam. Elongation factor Ts (EF-Ts) Ab was obtained from Primm Biotech as previously described (9), while the anti-Gapdh (FL-335) Ab was purchased from Santa Cruz Biotechnology. Halt protease and phosphatase inhibitor cocktail were obtained from Thermo Fisher Scientific. Endotoxin-free recombinant mouse IL-1α and IL-1β proteins were purchased from BioLegend.

### Bacterial strains, cell culture, and infection.

Vero76 cells (an African green monkey kidney line; ATCC, RL-1587) were maintained in minimal Dulbecco’s modified Eagle’s medium (DMEM) supplemented with 10% heat-inactivated fetal bovine serum (FBS) at 37°C with 5% CO_2_. R. montanensis strain M5/6 and *R. rickettsia* strain Sheila Smith were obtained from Ted Hackstadt (Rocky Mountain Laboratories, NIH, MT, USA), and R. typhi strain Wilmington was obtained from the CDC. All *Rickettsia* strains were propagated in Vero76 cells grown in DMEM supplemented with 5% FBS at 34°C with 5% CO_2_. All *Rickettsia* cells were purified as previously described (7). For infection of BMDMs, purified *Rickettsia* spp. were used at a multiplicity of infection (MOI) of 50, to ensure the presence of enough bacteria at early stage of infection, for host response (5, 8, 9). For infection using heat-inactivated bacteria, purified *Rickettsia* spp. were heated at 90°C for 20 min (42). Rickettsiosis in mice was induced by tail vein injection (i.v.) of purified *Rickettsia* cells (10^5^ to 10^6^ PFU) resuspended in PBS. At days 1, 3, and 7 after administration, blood was collected, and serum cytokine levels were measured by flow cytometry. Splenic tissue specimens were collected at the indicated times and used for bacterial burden analysis by quantitative PCR (qPCR) as described below.

### Differentiation of bone marrow-derived macrophages.

Bone marrow cells were isolated from femurs and tibias of WT, *Il-1β^−/−^*, *Il-1α^−/−^*, *Gsdmd^−/−^*, *Casp-1^−/−^*, *Casp-11^−/−^*, and *Casp-1/11^−/−^* mice. Femurs from *Casp-1^−/−^*, *Casp-11^−/−^*, and *Casp-1*/*11^−/−^* mice were kindly provided by Amal Amer (The Ohio State University, OH, USA), while bones from *Il-1β^−/−^* or *Il-1α^−/−^* mice were obtained from Thirumala-Devi Kanneganti (St. Jude Children’s Research Hospital, TN, USA). Femurs from *Gsdmd^−/−^* were kindly provided by Matthew Welch (University of California, Berkeley, CA, USA). Differentiation was induced by culturing bone marrow cells in RPMI 1640 medium supplemented with 10% FBS and 30% L929-conditioned medium (a source of macrophage colony-stimulating factor) with culture for 7 days as described previously (43).

### Measurement of cytokines and chemokines.

IL-1 cytokine concentrations in the sera of mice or supernatants from cultured BMDMs were assessed using the Legendplex mouse inflammation kit (BioLegend) following the manufacturer’s instructions as described previously (43).

### RNA isolation and quantitative real-time PCR.

BMDM samples were collected at 2, 6, 24, and 48 h postinfection, while spleens were collected at day 3 or 7 postinfection. RNA was extracted from 1 × 10^6^ BMDMs or 100 μL of organ homogenate using the Quick-RNA miniprep kit (ZymoResearch). The iScript reverse transcription supermix kit (Bio-Rad; 1708841) was used to synthesize cDNAs from 200 ng of RNA according to the manufacturer's instructions. Quantitative real-time PCR (qRT-PCR) was performed using SYBR green (Thermo Fisher Scientific) and 2 μL cDNA, and for the rickettsial citrate synthase gene (*GltA*), 1 μM each oligonucleotides 5′-CATAATAGCCATAGGATGAG-3′ (forward [F]) and 5′-ATGATTTATGGGGAACTACC-3′ (reverse [R]) were used and the results analyzed as described previously (25). Oligonucleotides for *Gapdh* were obtained from Qiagen.

### Extract preparation and Western blot analysis.

*Rickettsia*-infected BMDM cells were lysed for 2 h at 4°C in ice-cold lysis buffer (50 mM HEPES [pH 7.4], 137 mM NaCl, 10% glycerol, 1 mM EDTA, and 0.5% NP-40, supplemented with protease and phosphatase inhibitory cocktails) as described previously (43). Equal amounts of protein were loaded for SDS-PAGE, and membranes were probed with anti-Casp-1, anti-Casp-11, anti-Gsdmd, anti-IL-1α, anti-IL-1β, anti-EF-Ts, and anti-Gapdh Abs, followed by enhanced chemiluminescence with secondary Abs conjugated to horseradish peroxidase.

### Neutralization of endogenous IL-1α and IL-1β.

For *in vivo* neutralization of IL-1α and IL-1β, C57BL/6J WT mice were i.v. injected with 250 μg of anti-IL-1α (clone ALF-161; BioXCell), anti-IL-1β (clone B122; BioXCell), or an IgG isotype control (Armenian hamster IgG; BioXCell) Ab 24 h before the induction of mild rickettsiosis using 10^5^ PFU of R. typhi, R. rickettsii, or R. montanensis.

### Adoptive transfer of bone marrow-derived macrophages.

C57BL/6J WT mice were injected (i.v.) twice with PBS- or dichloromethylene biphosphate (Cl_2_MBP)-liposomes 72 and 48 h prior to macrophage transfer as described previously (43). Next, C57BL/6J WT mice were injected (i.v.) with WT, *Il-1β^−/−^*, or *Il-1α^−/−^* BMDMs (5 × 10^6^ cells/mouse), followed by infection (24 h post-Mϕ transfer) with R. typhi, R. rickettsii, R. montanensis, or PBS (dose of 10^5^ PFU).

### Statistical analysis.

Endpoint studies of mice subjected to mild (10^5^ PFU) and severe (10^6^ PFU) rickettsiosis were analyzed by using Kaplan-Meir survival curves and the log-rank test (GraphPad Prism Software, version 8). The statistical significance was assessed using analysis of variance (ANOVA) with Tukey’s multiple-comparison posttest (GraphPad). Data are presented as the mean ± standard error of the mean (SEM), unless stated otherwise. The alpha level was set to 0.05.
